# Third-Generation High-Vision Ultrathin Endoscopy Using Texture and Color Enhancement Imaging and Narrow-Band Imaging to Evaluate Barrett’s Esophagus

**DOI:** 10.3390/diagnostics12123149

**Published:** 2022-12-13

**Authors:** Mitsushige Sugimoto, Yusuke Kawai, Yoshika Akimoto, Mariko Hamada, Eri Iwata, Masaki Murata, Hitomi Mizuno, Ryota Niikura, Naoyoshi Nagata, Masakatsu Fukuzawa, Takao Itoi, Takashi Kawai

**Affiliations:** 1 Department of Gastroenterological Endoscopy, Tokyo Medical University Hospital, Tokyo 160-0023, Japan; 2 Department of Gastroenterology, National Hospital Organization Kyoto Medical Center, Kyoto 612-8555, Japan; 3 Toyoda Aoba Clinic, Shizuoka 438-0821, Japan; 4 Department of Gastroenterology and Hepatology, Tokyo Medical University Hospital, Tokyo 160-0023, Japan

**Keywords:** texture and color enhancement imaging, narrow-band imaging, ultrathin endoscopy, Barrett’s esophagus, reflux esophagitis

## Abstract

It remains unclear whether texture- and color-enhancement imaging (TXI) and narrow-band imaging (NBI) provide an advantage over white-light imaging (WLI) in Barrett’s esophagus. We compared endoscopic findings and color differences between WLI and image-enhanced endoscopy (IEE) using a third-generation ultrathin endoscope. We retrospectively enrolled 40 patients who evaluated Barrett’s esophagus using WLI, TXI, and NBI. Color differences determined using the International Commission on Illumination 1976 (L∗, a∗, b∗) color space among Barrett’s epithelium, esophageal, and gastric mucosa were compared among the endoscopic findings. As the secondary outcome, we assessed the subjective visibility score among three kinds of endoscopic findings. The prevalence of Barrett’s esophagus and gastroesophageal reflux disease (GERD) in WLI was 82.5% and 47.5%, respectively, and similar among WLI, TXI, and NBI. Color differences between Barrett’s epithelium and esophageal or gastric mucosa on NBI were significantly greater than on WLI (all *p* < 0.05). However, the color difference between Barrett’s epithelium and esophageal mucosa was significantly greater on NBI than TXI (*p* < 0.001), and the visibility score of Barrett’s epithelium detection was significantly greater on TXI than NBI (*p* = 0.022), and WLI (*p* = 0.016). High-vision, third-generation ultrathin endoscopy using NBI and TXI is useful for evaluating Barrett’s epithelium and GERD compared with WLI alone.

## 1. Introduction

Barrett’s esophagus, related to esophageal adenocarcinoma, is clinically recognized as requiring routine and careful endoscopic surveillance in both Western and Asian countries [1,2]. Among the various types, long-segment Barrett’s esophagus (LSBE) involves more than 3 cm of Barrett’s epithelium at the esophagocardial (EC) junction and is well known as a major risk factor for esophageal adenocarcinoma. In Japan, the prevalence of Barrett’s esophagus and LSBE among patients who undergo endoscopy following a routine health check-up is 56.2% and less than 1%, respectively [1,3]. The Japan Esophageal Society defines the esophagogastric junction (EGJ) as the distal margin of the palisade vessels of the lower esophagus, and the columnar-appearing area of mucosa between the squamocolumnar junction and EGJ as Barrett’s epithelium [4]. Thus, appropriate evaluation of the palisade vessels during routine endoscopy is important. White-light imaging (WLI) is currently the most common endoscopic evaluation method for Barrett’s esophagus, and recent advances in endoscopy equipment have facilitated the detection of palisade vessels by WLI. Nevertheless, WLI does not endoscopically detect Barrett’s epithelium in all patients with Barrett’s esophagus [3,5].

Recent advances in image-enhanced endoscopy (IEE), including narrow-band imaging (NBI), blue laser imaging (BLI), and linked color imaging (LCI), have improved the detection rate of gastric cancer and intestinal metaplasia [6,7,8,9,10,11,12], esophageal adenocarcinoma [13,14], and Barrett’s epithelium [3,5]. Among IEEs, texture and color enhancement imaging (TXI), which utilizes Retinex theory-based image processing technology to enhance three imaging factors in WLI (texture, brightness, and color), which facilitates the clear definition of subtle tissue differences (e.g., normal mucosa and neoplasm) [15,16,17]. TXI can selectively enhance brightness in dark areas of an endoscopic image and subtle tissue differences, such as slight morphological or color changes, while simultaneously preventing over-enhancement [18]. However, it is unclear whether endoscopic observation using TXI more clearly reveals the presence of palisade vessels, as well as the area of Barrett’s epithelium and reflux esophagitis, compared to WLI.

Here, we investigated whether third-generation, ultrathin endoscopy with TXI or NBI improves the visibility of Barrett’s esophagus and reflux esophagitis compared with standard WLI.

## 2. Materials and Methods

### 2.1. Study Design and Patients

This study was conducted as a retrospective cohort study at Tokyo Medical University Hospital to investigate the efficacy of TXI and NBI with third-generation, high-vision, ultrathin endoscopy to evaluate Barrett’s esophagus and reflux esophagitis. We enrolled 40 patients aged ≥20 years who underwent third-generation, high-vision, ultrathin endoscopy. The patients were evaluated as having Barrett’s esophagus and reflux esophagitis using three kinds of endoscopic findings (WLI, TXI, and NBI). We did not perform the pathological evaluation for Barrett’s esophagus. Exclusion criteria were a history of esophageal and gastric surgery and a lack of clear images with which to evaluate endoscopic Barrett’s esophagus and reflux esophagitis. All patients enrolled in this study overlapped those in our previous report on the efficacy of IEE, compared to WLI, for detecting gastric atrophy and intestinal metaplasia [11]. The study protocol adhered to the ethical principles of the Declaration of Helsinki and was approved by the institutional review board of Tokyo Medical University.

### 2.2. Endoscopy and Evaluation of Reflux Esophagitis and Barrett’s Esophagus

**Figure 1:**
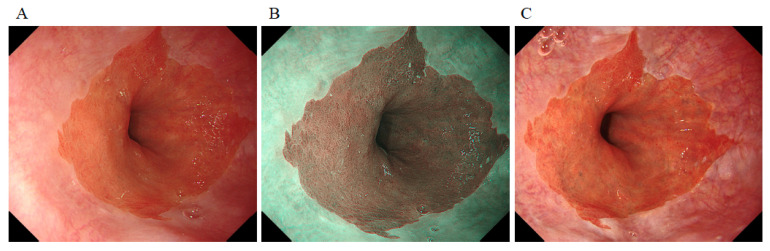
Images taken using a third-generation ultrathin endoscope by white-light imaging (**A**), narrow-band imaging (**B**), and texture and color enhancement imaging (**C**).

Endoscopy was performed using a third-generation, high-vision GIF-1200N ultrathin endoscope with the EVIS X1 system (Olympus Co., Tokyo, Japan). Barrett’s esophagus and reflux esophagitis were evaluated using WLI, NBI, and TXI (Figure 1A–C).

The presence of esophageal mucosal injury was assessed according to the Los Angeles classification (Grades A to D) [19]. Grade M was defined as mucosal findings of reddish or whitish turbidity [20]. Barrett’s esophagus was endoscopically diagnosed by the appearance of columnar-appearing mucosa of >5 mm in length around the lower esophagus. Hiatal hernia was endoscopically diagnosed when the EG junction was dislocated toward the esophageal site by more than 2 cm [21].

Expert endoscopists, who were certified as endoscopists by the Japan Endoscopic Society, independently evaluated Barrett’s esophagus using WBI, NBI, and TXI after endoscopy. They were blinded regarding both the diagnosis and clinical information.

### 2.3. Color Measurement among Barrett’s Epithelium, Esophageal and Gastric Mucosa

We randomly selected three pairs of each site of Barrett’s esophagus, esophageal and gastric mucosa (3 points of Barret esophagus vs. 3 points of gastric mucosa, and 3 points of Barret’s esophagus vs. 3 points of esophageal mucosa), and calculated the color difference, using three pairs in each patient, as previously reported [11]. A region of interest (ROI) was selected in the 2 mm inside Barrett’s esophagus and surrounding 2 mm outside esophageal, or gastric mucosa, on the endoscopic image. Each sample area (Barrett’s esophagus, esophageal, and gastric mucosa) at the EC junction was continuously imaged by WLI, NBI, and TXI with a similar composition. Color differences were calculated using the International Commission on Illumination (CIE) 1976 (L*, a*, b*) color space [11,22,23]. A color difference was defined as ΔE, which expresses the distance between two points in the color space. ΔE was calculated using the following formula: {(ΔL*)^2^ + (Δa*)^2^ + (Δb*)^2^}^1/2^. Each ΔL*, Δa*, and Δb* value was determined by a computer operator, who was blinded to clinical information, using Adobe Photoshop, version 22.5.1 (Adobe KK, Tokyo, Japan).

### 2.4. Evaluation of Reflux-Related Symptoms

Gastroesophageal reflux disease (GERD)-related symptoms were evaluated using the Frequency Scale for the Symptoms of GERD (FSSG) [24,25] and the IZUMO scale [26]. A total score of ≥8 in the FSSG indicates probable GERD [24,25]. The 12 items of the FSSG are often classified into 2 domains: a reflux-related symptom domain and an acid-related dysmotility symptom domain.

### 2.5. Visibility Assessment of Barrett’s Esophagus Observed by WLI, NBI, and TXI

To assess the subjective differences in the color tone of Barrett’s esophagus by WLI, NBI, and TXI, a visibility assessment was performed by 6 endoscopists. The endoscopic images were selected views obtained under almost the same conditions. The 60 endoscopic images (20 cases including WLI, NBI, and TXI) were randomly ordered and displayed, and the evaluators independently assessed them in a single session. The endoscopists scored the color tone of Barrett’s esophagus based on a 4-point visibility scale. Visibility scores were defined as follows: 4, excellent visibility (easily detectable); 3, good visibility (detectable with careful observation); 2, fair visibility (hardly detectable without careful examination); and 1, poor visibility (not detectable without repeated careful examination).

### 2.6. Statistical Analysis

Parameters including age, height, body weight, and FSSG, IZUMO scale, and GSRS questionnaire scores are expressed as mean ± standard deviation (SD). Categorical variables for Barrett’s esophagus and reflux esophagitis among WLI, NBI, and TXI were summarized as n (%) and compared using χ^2^ tests. Statistically significant differences in mean questionnaire scores and mean ΔE*, ΔL*, Δa*, and Δb* among WLI, NBI, and TXI were determined using Student’s *t*-test. A *p*-value < 0.05 was considered statistically significant, and all *p*-values were two sided. All statistical analyses were performed using the statistical analysis software SPSS, version 27.0 (IBM Japan, Tokyo, Japan).

When we set a 0.50 effect size, 0.05 alpha error, 0.80 sample power (1-beta), and allocation ratio of 1 for differences between two dependent means (matched pairs) by the *t*-test, the sample number required is 34 patients [G*Power software (ver. 3.1.9.6), Heinrich-Heine-Universität Düsseldorf, Düsseldorf, Germany]. Therefore, we enrolled 40 patients in this study.

## 3. Results

### 3.1. Patient Characteristics

Among patients who underwent endoscopy using a GIF-1200N ultrathin endoscope with the EVIS X1 system, we enrolled 40 patients who were evaluated as having Barrett’s esophagus and reflux esophagitis by WLI, NBI, and TXI. The mean age was 74.2 ± 5.8 years, and 62.5% of patients were males (Table 1). Baseline diseases included peptic ulcer in 5.0% of patients [number (n) = 2] and gastric cancer in 5.0% (n = 2). Drugs taken included proton pump inhibitors (PPIs) in 27.5% (n = 11).

In the analysis of reflux-related symptoms, the mean acid-related symptom score and total score of the F-scale questionnaire were 2.3 ± 3.0 and 4.3 ± 4.8, respectively (Table 1). The prevalence of patients with a total F-scale score of ≥8 was 25.0% (10/40), indicating probable GERD.

### 3.2. Barrett’s Esophagus and Reflux Esophagitis Using WLI, NBI, and TXI

**Figure 2:**
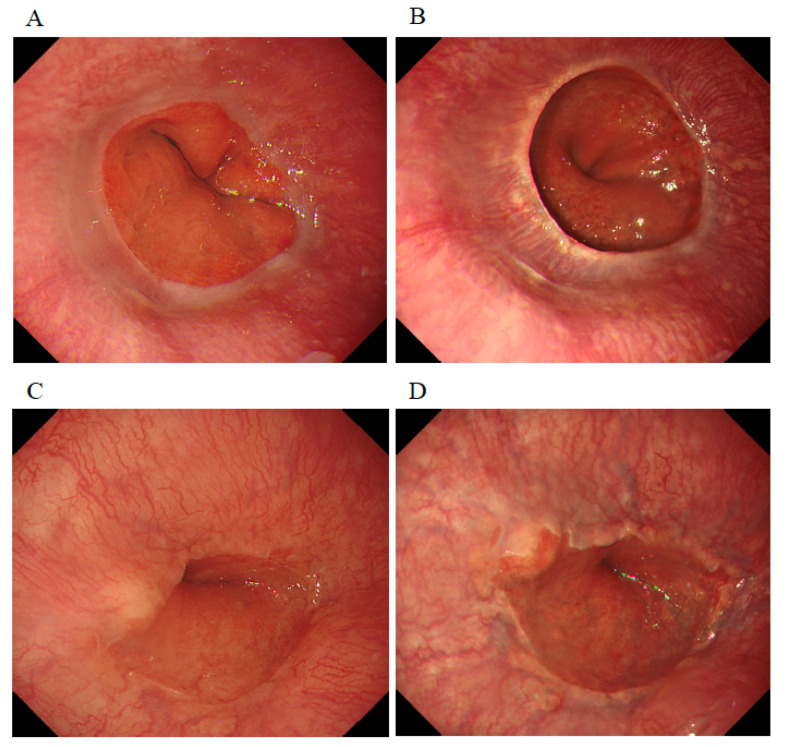
Typical endoscopic images in patients in which texture and color enhancement imaging (TXI) clearly showed GERD Grade M, whitish turbidity, in the esophagocardial junction compared to white-light imaging (WLI). Case 1, WLI (**A**) and TXI (**B**) and Case 2, WLI (**C**) and TXI (**D**).

The prevalence of short-segment Barrett’s esophagus (SSBE), LSBE, GERD, and reflux esophagitis in WLI was 82.5%, 0%, 47.5%, and 5.0%, respectively (Table 2). Although the prevalence of GERD with TXI was 62.5%, and thus higher than that with WLI (47.5%) and NBI (40.0%), this difference was not significant (*p* = 0.137). Further, there were no significant differences in rates of SSBE or GERD among WLI, TXI, and NBI. Of 40 patients, only 1 patient was diagnosed with Barrett’s epithelium by only TXI in this study. Of 13 patients with GERD grade M (whitish turbidity in the EC junction) evaluated by WLI, four cases were more clearly observed on TXI than on WLI (Figure 2).

### 3.3. Color Differences between SSBE and Gastric or Esophageal Mucosa

The color differences (ΔE*) between esophageal and gastric mucosa were 15.7 ± 6.9 in WLI, 33.4 ± 11.8 in NBI, and 18.2 ± 8.3 in TXI, respectively (Table 3). Although the color difference between esophageal and gastric mucosa was similar between WLI and TXI, this difference was significantly greater in NBI (*p* < 0.001 and *p* < 0.001).

When color differences were compared between esophageal mucosa and Barrett’s epithelium, NBI was significantly greater than in WLI and TXI (*p* <0.001 and <0.001, respectively). However, there was no significant difference between WLI and TXI (Table 3).

The color differences between gastric mucosa and Barrett’s epithelium were 6.7 ± 3.3 in WLI, 10.8 ± 7.6 in NBI, and 8.8 ± 4.9 in TXI, respectively. Although the difference was similar between NBI and TXI (*p* = 0.212), these were significantly greater than in WLI (*p* = 0.014 and 0.049, respectively).

**Figure 3:**
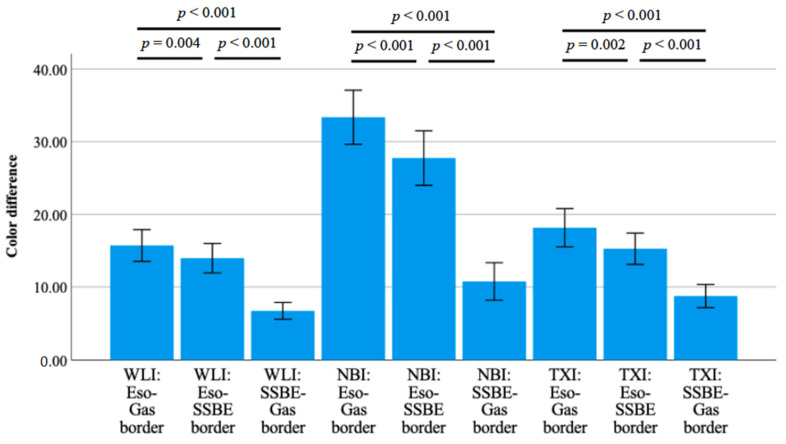
Color differences among short-segment Barrett’s esophagus, esophageal, and gastric mucosa with three kinds of detection methods using third-generation high-vision GIF-1200N. Color differences between SSBE and esophageal or gastric mucosa in all detection methods were significantly smaller (*p* < 0.001 and < 0.001). Abbreviations: NBI: narrow-band imaging, SSBE: short-segment Barrett’s esophagus, TXI: texture and color enhancement imaging, WLI: white-light imaging.

With regard to the color differences between esophageal and gastric mucosa, those between Barrett’s epithelium and esophageal or gastric mucosa were significantly smaller in each detection method (Table 3 and Figure 3).

### 3.4. Visibility Assessment of Barrett’s Esophagus

The mean ± SD of the visibility scores of Barrett’s esophagus detection evaluated by six endoscopists were significantly higher for TXI (3.4 ± 0.4) than for WLI (2.9 ± 0.8, *p* = 0.016) and NBI (3.0 ± 0.4, *p* = 0.022) (Table 4). The visibility scores between gastric mucosa and Barrett’s esophagus were significantly higher for TXI (2.6 ± 0.5) than for NBI (2.2 ± 0.2, *p* = 0.002).

## 4. Discussion

The combination of third-generation high-vision ultrathin endoscopy and a new processor allows NBI to reveal significantly greater color differences for the differentiation of Barrett’s epithelium from esophageal mucosa and gastric mucosa than WLI, and allows TXI to reveal color differences between Barrett’s epithelium and gastric mucosa. In this study, although we found that the detection of Barrett’s epithelium and evaluation of the severity of reflux esophagitis were similar among WLI, NBI, and TXI, the visibility of Barrett’s esophagus detection evaluated by six endoscopists was significantly higher for TXI than for WLI and NBI. This observation suggests that although TXI and NBI make it easier to diagnose Barrett’s epithelium endoscopically by increasing the color difference and visibility, diagnosis with WLI is nevertheless possible. Therefore, evaluation using third-generation ultrathin endoscopy with NBI and TXI appears useful for identifying patients with Barrett’s esophagus.

### 4.1. Efficacy of NBI in Identifying Barrett’s Esophagus

Barrett’s esophagus and reflux esophagitis are possible risk factors for esophageal adenocarcinoma, and a conclusive diagnosis of Barrett’s esophagus and reflux esophagitis during endoscopic surveillance is important. A position statement of the European Society of Gastrointestinal Endoscopy recommends the endoscopic surveillance of Barrett’s esophagus by inspection with high-definition WLI, followed by random biopsy of Barrett’s epithelium (every 1 to 2 cm throughout Barrett’s esophagus segment) in the absence of any lesions [27]. However, this random biopsy method has drawbacks: the area of biopsied tissue sampled accounts for less than 5% of the total area of Barrett’s esophagus, meaning that focal dysplasia can be missed, especially in patients with LSBE [28]. Moreover, despite its high definition, WLI does not endoscopically detect Barrett’s epithelium in all patients with Barrett’s epithelium and esophageal cancer [3,5]. Several IEEs have been developed accordingly to improve visualization (e.g., mucosal surface and vascular microstructures) of Barrett’s epithelium, focal dysplasia, and esophageal adenocarcinoma [6,7,8,9,10]. In fact, a recent meta-analysis by the American Society for Gastrointestinal Endoscopy showed that the pooled sensitivity, negative predictive value, and specificity during endoscopic surveillance of Barrett’s esophagus using NBI were 94.2% [95% Confidence Interval (CI) 82.6–98.2], 97.5% (95% CI; 95.1–98.7), and 94.4% (95% CI; 80.5–98.6), respectively [29]. A working group of the Barrett’s International NBI Group recently created a new simplified international classification for findings on NBI combined with magnifying endoscopy. An international multicenter trial showed that this classification had high diagnostic accuracy and good inter-observer agreement in the diagnosis of esophageal adenocarcinoma [30]. Therefore, surveillance endoscopy for Barrett’s esophagus and reflux esophagitis using NBI, BLI, and LCI is considered to be an acceptable method worldwide.

### 4.2. Efficacy of TXI in Identifying Barrett’s Esophagus and Reflux Esophagitis

TXI was launched as a new optical IEE modality in 2020 with the expectation that it would provide a better detection rate of gastrointestinal diseases than WLI by selectively enhancing brightness in dark areas of an endoscopic image and subtle tissue differences [15,16,17,18,31,32]. Ishikawa et al. [17] reported that the color difference surrounding gastric atrophic borders and gastric cancer borders was significantly greater in TXI than in WLI (atrophy: 14.2 ± 8.0 vs. 8.7 ± 4.2, *p* < 0.01; gastric cancer: 18.7 ± 16.0 vs. 8.0 ± 4.2, *p* < 0.01). Abe et al. [32] reported that the visibility score of gastric cancer was improved in 35% and 20% of cases in TXI mode 1 and TXI mode 2, respectively, when compared to WLI, especially in patients with macroscopic type 0-IIc or 0-IIb in TXI mode 1. We previously demonstrated that TXI and NBI also produce significantly larger differences in the tissue surrounding intestinal metaplasia as a precancerous lesion of gastric cancer than WLI, in addition to greater color differences in areas surrounding atrophic borders [11]. To date, however, it has remained unclear whether TXI reveals esophageal disease, including Barrett’s epithelium and reflux esophagitis, more clearly than WLI. Dobashi et al. [16] reported that the mean color difference values between squamous cell carcinoma and surrounding esophageal mucosa were 11.6 ± 6.8 in WLI, 18.6 ± 10.8 in TXI mode 1, 15.7 ± 10.3 in TXI mode 2 and 18.8 ± 12.0 in NBI, and that the color differences of TXI mode 1, TXI mode 2, and NBI were significantly higher than those of WLI (*p* < 0.001). Although we are unaware of any study reporting the usability of TXI in detecting Barrett’s epithelium and GERD, here, we demonstrate the advantages of TXI in the observation of patients with GERD grade M, especially in those with whitish turbidity in the EC junction. In addition, although the color difference between Barrett’s epithelium and gastric mucosa was similar between NBI and TXI, differences in NBI and TXI were significantly greater than those in WLI. This suggests that TXI, as evaluated by other IEEs, may improve the visibility and detectability of Barrett’s epithelium. Although our present study suggests that surveillance endoscopy using TXI has an advantage over WLI in revealing color differences between Barrett’s epithelium and gastric mucosa, it was conducted in a small number of patients, and is preliminary. Therefore, we plan to conduct a prospective trial that can prove the efficacy of a combination of WLI and TXI with or without magnifying endoscopy in the detection of Barrett’s esophagus in the near future.

### 4.3. Usefulness of Third-Generation High-Vision Ultrathin Endoscopy

Endoscopic examination in Japan is often performed transnasally to reduce invasiveness and distress to the patient, especially in health check-ups and private clinic settings [33,34]. Although previous generations of this technology had several disadvantages, including the need for complex planning, poor image quality, and a lower disease detection rate [35], third-generation ultrathin endoscopy with a new high-quality complementary metal-oxide semiconductor sensor provides markedly improved image quality. In fact, color differences surrounding atrophy produced by NBI on the third-generation GIF-1200N endoscope were significantly greater than those on second-generation GIF-290N (19.2 ± 8.5 vs. 14.4 ± 6.2, *p* = 0.001) [36]. An increase in health check-ups is leading to a parallel rise in the conduct of transnasal endoscopy tests, making it prudent to evaluate the usefulness of not only standard oral endoscopy, but also these tests. However, to date, reports investigating the effectiveness of IEE for objectively evaluating gastrointestinal diseases, including Barrett’s esophagus, using high-vision ultrathin endoscopy were limited [11]. Therefore, we evaluated Barrett’s esophagus using ultrathin endoscopy but not standard endoscopy. Combining WLI and IEE using third-generation high-vision ultrathin endoscopy is expected to become increasingly important in surveillance endoscopy to identify esophageal cancer, especially in health check-ups and private clinics.

Artificial intelligence (AI) has recently shown dramatic developments in the field of gastrointestinal endoscopy [37]. Because AI is a promising tool that can reduce the burden of a time-consuming endoscopic image review in cancer screening, while ensuring quality, and might also support endoscopists, AI-aided diagnosis is being utilized for cancer detection. We look forward to the development of AI systems using IEE, such as NBI and TXI, for the detection of Barrett’s esophagus, esophageal dysplasia, cancer, and reflux esophagitis and their evaluation in multicenter prospective trials.

### 4.4. Limitations

This study has a few limitations that warrant mentioning. First, it was conducted on a pilot basis, and the sample size was small. Second, it was a single-center retrospective study. Third, although the pathological examination is considered the gold standard for the evaluation of Barrett’s epithelium, we did not have pathological data. However, a recent meta-analysis showed that the pooled sensitivity, negative predictive value, and specificity during endoscopic surveillance of Barrett’s esophagus reached more than 94%, [29], suggesting that endoscopic evaluation of Barrett’s esophagus may be acceptable compared with pathological examination.

## 5. Conclusions

We showed that NBI and TXI with a third-generation ultrathin endoscope enhanced color differences surrounding Barrett’s esophagus according to the CIE 1976 (L*a*b*) color space compared to WLI, and that the visibility of Barrett’s esophagus detection evaluated were significantly higher for TXI than for WLI and NBI. Given that advances in endoscopic technology have markedly enhanced the diagnostic capability of endoscopy, it is important to identify the best diagnostic method for Barrett’s esophagus. TXI has the potential for the sensitive and real-time detection of Barrett’s esophagus via its ability to enhance slight depressions and elevations and highlight color differences. Large-scale multicenter prospective studies are needed to investigate the efficacy of TXI and NBI with high-vision ultrathin endoscopy and new processors for detecting esophageal adenocarcinoma.

## Figures and Tables

**Table 1:** Characteristics of patients enrolled in this study.

	All Patients (n = 40)
Age (years ± SD)	74.2 ± 5.8
Sex [male, n (%)]	25 (62.5%)
Height (cm ± SD)	161.3 ± 9.3
Body weight (kg ± SD)	59.3 ± 10.6
*H. pylori* infection, negative/current/eradicated [n/n/n]	3/0/37
Smoking, never/current/past [n/n/n]	26/1/13
Alcohol [n (%)]	26 (65.0%)
Diseases	
Peptic ulcer [n (%)]	2 (5.0%)
Gastric cancer [n (%)]	2 (5.0%)
Drugs	
PPI [n (%)]	11 (27.5%)
Antiplatelet drugs [n (%)]	12 (30.0%)
Anticoagulants [n (%)]	4 (10.0%)
Bisphosphonates	2 (5.0%)
Abdominal symptoms	
F-scale questionnaire	
Acid-related symptom score	2.3 ± 3.0
Dysmotility-related symptom score	2.0 ± 2.1
F-scale total score	4.3 ± 4.8
IZUMO-scale questionnaire	
Reflux	3.3 ± 4.6
Pain	1.1 ± 1.7
Fullness	0.7 ± 1.1
IZUMO scale total score	4.1 ± 4.7

Abbreviations: *H. pylori*: *Helicobacter pylori*, SD: standard deviation, PPI: proton-pump inhibitor.

**Table 2:** Endoscopic Barrett’s esophagus and esophagitis among WLI, NBI, and TXI.

	WLI	NBI	TXI	*p* Value
Barrett’s esophagus [n (%)]	33 (82.5%)	33 (82.5%)	36 (90.0%)	0.596
GERD [n (%)]	19 (47.5%)	16 (40.0%)	25 (62.5%)	0.137
Grade MW [n (%)]	13 (32.5%)	10 (25.0%)	16 (40.0%)	0.996
Grade MR [n (%)]	4 (10.0%)	4 (10.0%)	7 (17.5%)	
Grade A [n (%)]	1 (2.5%)	1 (2.5%)	1 (2.5%)	
Grade B [n (%)]	1 (2.5%)	1 (2.5%)	1 (2.5%)	

Abbreviations: MR: grade M (reddish), MW: grade M (whitish turbidity), NBI: narrow-band imaging, TXI: texture and color enhancement imaging, WLI: white-light imaging.

**Table 3:** Color differences among esophageal mucosa, gastric mucosa, and SSBE.

		WLI	NBI	TXI	*p* Value		
					WLI vs. NBI	NBI vs. TXI	TXI vs. WLI
Esophageal mucosa	ΔL*	9.6 ± 9.0	24.1 ± 13.6	12.7 ± 8.7	<0.001	<0.010	0.062
(vs. gastric mucosa)	Δa*	−3.3 ± 6.0	−20.0 ± 8.2	−5.8 ± 7.9	<0.001	<0.001	0.045
	Δb*	−7.6 ± 4.3	−3.3 ± 3.9	−6.8 ± 4.9	<0.001	0.002	0.380
	ΔE*	15.7 ± 6.9	33.4 ± 11.8	18.2 ± 8.3	<0.001	<0.001	0.069
Esophageal mucosa	ΔL*	7.9 ± 7.7	22.0 ± 11.5	9.3 ± 7.2	<0.001	<0.001	0.305
(vs. Barrett’s esophagus)	Δa*	−4.0 ± 4.4	−15.2 ± 6.2	−5.6 ± 6.5	<0.001	<0.001	0.243
	Δb*	−7.7 ± 3.9	−2.1 ± 2.2	−7.1 ± 4.0	<0.001	<0.001	0.662
	ΔE*	14.0 ± 5.8 ^#^	27.7 ± 11.1 ^#^	15.3 ± 6.6 ^#^	<0.001	<0.001	0.390
Gastric mucosa	ΔL*	−2.1 ± 6.0	−2.5 ± 9.8	−3.6 ± 7.1	0.336	0.426	0.415
(vs. Barrett’s esophagus)	Δa*	−0.4 ± 3.5	5.8 ± 5.6	−0.3 ± 5.3	<0.001	<0.001	0.482
	Δb*	−0.2 ± 2.2	1.7 ± 2.6	−0.5 ± 3.4	0.008	0.009	0.801
	ΔE*	6.7 ± 3.3 ^#,$^	10.8 ± 7.6 ^#,$^	8.8 ± 4.9 ^#, $^	0.014	0.212	0.049

Abbreviations: NBI: narrow-band imaging, WLI: white-light imaging, ΔL*: change in brightness, Δa*: change in red-green component, Δb*: change in yellow-blue component, ΔE*: color difference. #: <0.05 vs. color differences between esophageal mucosa and gastric mucosa. $: < 0.05 vs. color differences between esophageal mucosa and SSBE.

**Table 4:** Visibility assessment of Barrett’s esophagus.

	WLI	NBI	TXI	*p* Value		
				WLI vs. NBI	NBI vs. TXI	TXI vs. WLI
Detection of Barrett’s esophagus	2.9 ± 0.8	3.0 ± 0.4	3.4 ± 0.4	0.383	0.022	0.016
Border with Barrett’s esophagus and gastric mucosa	2.3 ± 0.6	2.2 ± 0.2	2.6 ± 0.5	0.075	0.002	0.072

Abbreviations: NBI: narrow-band imaging, TXI: texture and color enhancement imaging, WLI: white-light imaging. The endoscopists scored the color tone of Barrett’s esophagus based on a 4-point visibility scale. Visibility scores were defined as follows: 4, excellent visibility; 3, good visibility; 2, fair visibility; and 1, poor visibility.

## Data Availability

The data presented in this study are available on request from the corresponding author.
